# Combination therapy with docetaxel and S-1 as a first-line treatment in patients with advanced or recurrent gastric cancer: a retrospective analysis

**DOI:** 10.1186/1477-7819-8-40

**Published:** 2010-05-19

**Authors:** Kazuaki Tanabe, Takahisa Suzuki, Noriaki Tokumoto, Hideki Yamamoto, Kazuhiro Yoshida, Hideki Ohdan

**Affiliations:** 1 Department of Surgery, Division of Frontier Medical Science, Graduate School of Biomedical Science, Hiroshima University, Hiroshima, Japan; 2 Department of Surgical Oncology, Graduate School of Medicine, Gifu University, Gifu, Japan

## Abstract

**Background:**

We performed a single-institution retrospective study to evaluate the efficacy and toxicities of combination therapy with docetaxel and S-1 in patients with advanced or recurrent gastric cancer.

**Methods:**

Eighty-six patients with advanced or recurrent gastric cancer were enrolled. Patients received docetaxel, 40 mg/m^2^, on day 1 and oral S-1, 80 mg/m^2^/day, on days 1 to 14 every 3 weeks.

**Results:**

All 84 patients were assessable for response. The overall response rate was 52.4% (44/84) and the disease control rate was 96.4% (81/84). Median time to progression (TTP) and overall survival (OS) were 6.5 (95% CI, 4.8-8.1 months) and 15.1 months (95% CI, 11.7-18.5 months), respectively. The major toxicities were neutropenia, leukopenia, alopecia and anorexia. Grade 3 or 4 hematologic toxicities included neutropenia in 31 patients (36.0%), leukopenia in 27 (31.7%), febrile neutropenia in four (4.7%), and anemia in one (1.2%). Other grade 3 toxicities included anorexia in five patients (5.8%), and stomatitis, diarrhea and nausea in one each (1.2%). There was one treatment-related death (1.2%).

**Conclusion:**

The combination of docetaxel and S-1 had good clinical activity with acceptable toxicity in patients with advanced or recurrent gastric cancer.

## Introduction

Worldwide, gastric cancer ranks second among causes of all cancer-related deaths, with about 700,000 confirmed mortalities annually [1]. In Japan, gastric cancer is still the second most frequent cause of cancer-related death, despite advances in diagnosis and treatment. For patients with unresectable or recurrent gastric cancer, outcomes are extremely poor, with a median survival time, if untreated, of 3 to 5 months [2,3]. Many randomized controlled trials of various treatment regimens have been reported, including 5-fluorouracil, doxorubicin, and mitomycin (FAM) [4], epirubicin and cisplatin (CDDP) in combination with continuous infusion of 5-fluorouracil (ECF) [5], and 5-fluorouracil and cisplatin (FP) [6], but all produced median survivals of less than 1 year. No world-wide standard regimen has as yet been established.

Recently, two randomized controlled trials were reported from Japan [7,8]. One was the JCOG9912 trial, which showed S-1 to be non-inferior to continuous infusion of 5-fluorouracil with respect to overall survival (OS). Another was the SPIRITS trial, which revealed S-1 plus CDDP to be superior to S-1 alone with respect to OS. In clinical practice, S-1 plus CDDP has been recognized as the standard chemotherapy regimen for advanced or recurrent gastric cancer in Japan.

Docetaxel has shown promising activity in gastric cancer, both as monotherapy [9] and in combination with other agents [10-12]. We performed phase I and phase II studies of combination therapy with docetaxel and S-1 for patients with advanced or recurrent gastric cancer [13,14]. In the phase II study, the overall response rate was 56.3% (95% CI, 38-66%) and median survival time was 14.3 months (95% CI, 10.7-20.3 months). The most common severe toxicities were neutropenia (58.3%), leukopenia (41.7%), anorexia (14.6%) and stomatitis (8.3%). These findings suggested the regimen combining docetaxel with S-1 to be a promising first line therapy for advanced or recurrent gastric cancer. On the basis of this assumption, the objectives of the current study were to retrospectively clarify the efficacy and toxicities of the docetaxel and S-1 combination as a first-line treatment for patients with advanced or recurrent gastric cancer and to analyze prognostic factors in these patients.

## Patients and methods

### Patients

The subjects of this study were 86 patients treated between August 2001 and September 2009 at the Hiroshima University Hospital. Patients were eligible for this study if they had histologically confirmed advanced or recurrent gastric cancer, no prior therapy, including adjuvant therapy, Eastern Cooperative Oncology Group (ECOG) performance status <3, age ≧20 years, adequate organ function, and life expectancy of 3 months or more. Written informed consent was obtained from all patients prior to enrollment in the study according to institutional guidelines.

### Treatment regimen

S-1, at 80 mg/m^2^, was orally administered twice daily for 2 weeks, followed by a drug-free interval of 1 week (one cycle). The docetaxel infusion was started simultaneously with S-1 administration. Dexamethasone, 8 mg, was infused 1 hour before docetaxel administration. The dose of S-1 was reduced by 25% up to 50 mg/m2 in the event of any of the following toxicities during the previous treatment cycle: grade 4 leukopenia or neutropenia; thrombocytopenia ≧grade 3; and nonhematologic toxicity ≧grade 3 except anorexia, nausea, and vomiting. There were no dose reductions for docetaxel. Treatment with both S-1 and docetaxel was delayed for up to 3 weeks if patients had insufficient hepatic, cardiac, renal, or bone marrow function. (i.e., WBC <3,000/mm3, neutrophils <1,500/mm3, platelets <100,000/mm3, fever <38°C with grade 3 to 4 neutropenia, or nonhematologic toxicity ≧grade 3) Cycles were repeated every 3 weeks, and the treatment was continued until disease progression, unacceptable toxicity, or the patient refused further therapy.

### Evaluation of efficacy and toxicities

Responses were classified according to Response Evaluation Criteria In Solid Tumors (RECIST) guidelines [15]. Tumor size was measured by CT scan with a 5 mm slice thickness for all measurable lesions to assess responses every 4 to 6 weeks. Toxicity was graded according to Common Terminology Criteria for Adverse Events (CTCAE) version 3.0 [16].

### Statistical methods

OS was calculated from the date of chemotherapy initiation to the date of all-cause death or the latest follow-up. Time to progression (TTP) was calculated from the date of chemotherapy to the first day of disease progression. The median OS and TTP were estimated using the Kaplan-Meier method. Multivariate analysis of prognostic factors was performed by the Cox proportional hazard method to evaluate the influences of prognostic factors on patient survival. A *P *< 0.05 was considered to indicate a statistically significant difference.

## Results

### Patient characteristics

The characteristics of our patients are summarized in Table 1. Two patients were not evaluable for response; one patient had a treatment-unrelated early death, and the other refused the treatment for reasons not related to toxicity during the course of the 2nd cycle. Treatment administration of S-1 was delayed in 35 out of 633 cycles patients (range, 7-16 days) because of grade 3 or 4 neutropenia. No docetaxel doses were omitted. The median age was 63 years (range, 25-81), and 84 (93.0%) patients had good performance status (ECOG, 0 or 1). Seventy-one patients (82.6%) had advanced stage disease at diagnosis and 15 (17.4%) experienced relapse after curative surgery. A prior gastrectomy had been performed in 21 (24.4%) patients. The common major metastatic sites were lymph nodes (52.3%), the peritoneum (37.2%), and the liver (25.6%).

**Table 1:** Patient characteristics.

Characteristics	No. of patients (%)
Total no.	
Assemble for response	84^a ^(97.7)
Assemble for toxicity	86
Gender	
Male	58 (67.4)
Female	28 (32.6)
Age (years)	
Median	60 (80.2)
Range	25-81
Performance status by ECOG	
0	69 (80.2)
1	11 (12.8)
2	5 (5.8)
3	1 (1.2)
Disease status	
Advanced	71 (82.6)
Recurrent	15 (17.4)
Prior gastrectomy	
-	65 (75.6)
+	21 (24.4)
Metastatic site	
Liver	22 (25.6)
Lymph node	45 (52.3)
Peritoneum	32 (37.2)
Bone	3 (3.5)
Lung	2 (2.3)
Ovary	2 (2.3)
No. of organs involved	
1	63 (73.3)
2	21 (24.4)
3	2 (2.3)

^a ^Two parients were not evaluable.

### Tumor response and survival

**Figure 1:**
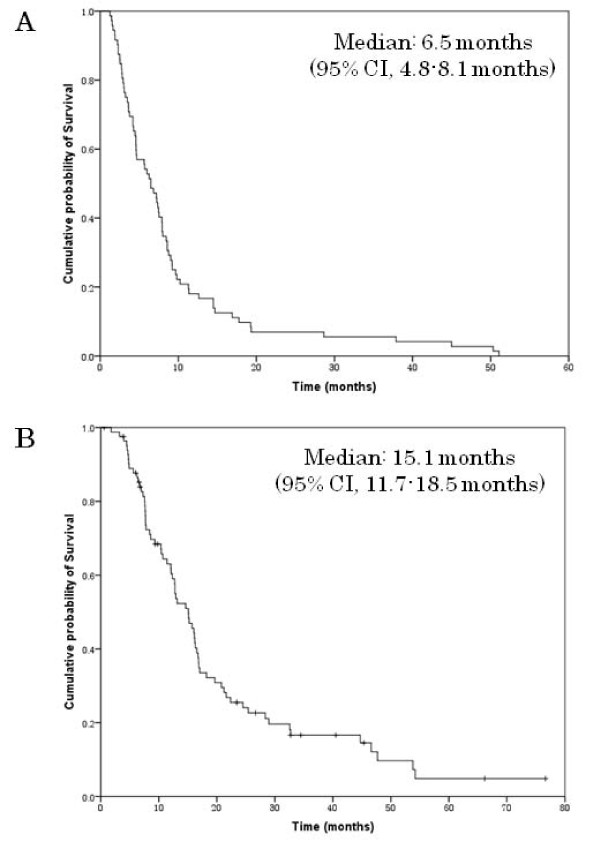
**The time to progression (A) and overall survival (B)**.

Eighty-two patients were available for the response evaluation. There were no patients showing complete response, 44 (52.4%) patients showing partial response (PR), 37 patients (44.0%) with stable disease (SD), and three (3.5%) who showed disease progression (PD) (Table 2). The overall response rate was 52.4% (95% confidence interval (CI), 42.9-64.5%). Fifty-two patients (60.5%) received second-line chemotherapy after failure of this regimen, including weekly paclitaxel and irinotecan plus cisplatin. At a median follow-up of 12.7 months, the median TTP was 6.5 months (95% CI, 4.8-8.1 months) (Fig. 1a), and the median OS was 15.1 months (95% CI, 11.7-18.5 months) (Fig. 1b).

**Table 2:** Response assessment.

	No. of patients	%
Complete response	0	0
Partial response	44	52.4
Stable disease	37	44.0
Progressive disease	3	3.6

The overall response rate was 52.4% (95% confidence interval, 42.9-64.5)

### Toxicities

In total, 633 cycles were administered. The median number of cycles administered per patient was six (range, 2-23). The toxicity profiles are summarized in Table 3. As to hematological toxicities, Grade 3 or 4 neutropenia was observed in 31 (36.0%) patients, leucopenia in 27 (31.7%) and anemia in one (1.2%). Grade 3 febrile neutropenia occurred in four (4.7%) patients. As to non-hematological toxicities, Grade 3 anorexia was observed in five (5.8) patients, and stomatitis, diarrhea, and nausea in one each (1.2%). Docetaxel and S-1 dosage reductions were necessary in 17 patients, because of Grade 4 neutropenia in 16 (18.6%) and Grade 3 diarrhea in one (1.2%). There was one treatment-related death (1.2%) in a patient who had sepsis. Grade 4 neutropenia was obserbed in this patient in the third cycle. The treatment of S-1 was discontinued while granulocyte colony-stimulating factor (G-CSF) and antibiotics were given. Despite intensive therapy, he died due to pneumonia progressed rapidly to sepsis.

**Table 3:** Hematologic and non-hematologic toxicities

	Grade of toxicities				% of Grade
		
Toxicity	1	2	3	4	3 or 4
Hematologic toxicities					
Leukopenia	8 (9.3)	10 (11.6)	25 (29.1)	2 (2.3)	31.4
Neutropenia	3 (3.5)	5 (5.8)	15 (17.4)	16 (18.6)	36.0
Anemia	8 (9.3)	1 (1.2)	1 (1.2)	-	1.2
Thrombocytopenia	5 (5.8)	-	-	-	-
Febrile neutropenia	-	-	4 (4.7)	-	4.7
Non-hematologic toxicities					
Alopecia	27 (31.4)	13 (15.1)	-	-	-
Anorexia	24 (27.9)	7 (8.1)	5 (5.8)	-	5.8
Diarrhea	8 (9.3)	3 (3.5)	1 (1.2)	-	1.2
Dysgeusia	6 (7.0)	-	-	-	-
Hyperpigmentation	12 (14.0)	-	-	-	-
Infection	-	-	-	1 (1.2)	1.2
Nausea	10 (11.6)	1 (1.2)	1 (1.2)	-	1.2
Stomatitis	14 (16.3)	2 (2.3)	1 (1.2)	-	1.2

Grading according to CTCAE (version 3.0)

### Prognostic factors

The results of univariate analyses of various patient and tumor variables are shown in Table 4. The estimated OS was significantly better for patients with good performance status, tumor response and second-line chemotherapy. In the Cox proportional hazard model, the only independent prognostic factor for OS was the tumor response (Table 5). Patients with partial response had significantly increased OS (Hazard ratio, 0.002 95% CI, 0.253-0.732; P = 0.002).

**Table 4:** Prognostic factor analysis (univariate).

	OS (months)	95% CI	*P *value
Age			
< median	15.2	11.5 - 19.0	0.491
≧ median	12.8	7.1 - 18.4	
Gender			
male	13.2	9.8 - 14.8	0.49
Female	16.8	10.7 - 22.8	
Performance status			
0-1	15.2	12.0 18.5	0.01
≧ 2	7.6	0 - 16.5	
Disease status			
Advanced	15.2	11.7 - 18.8	0.24
Recurrent	12.1	9.1 - 15.1	
Histology			
differentiated	14.5	9.1 - 18.2	0.357
undifferentiated	14.6	11.0 - 18.2	
No. of organs involved			
1	12.8	10.1 - 15.4	0.414
≧ 2	16.9	14.4 - 19.3	
Liver metastasis			
No	16.0	12.7 - 19.3	0.237
Yes	10.4	3.5 - 17.3	
Peritoneum metastasis			
No	15.1	9.5 - 20.7	0.54
Yes	14.6	11.1 - 18.1	
Tumor response			
No (SD or PD)	8.6	6.0 - 11.2	<0.001
Yes (PR)	18.2	12.7 - 23.7	
Second-line chemotherapy			
No	8.6	4.2 - 13.0	0.024
Yes	16.3	15.0 - 17.5	

OS, median overall survival

**Table 5:** Multivariate analyss of overall survival.

	*P *value	Hazard ratio	95% CI
Performance status	0.1	2.098	0.867 - 5.073
Tumor response	0.002	0.43	0.253 - 0.732
Second-line chemotherapy	0.573	0.855	0.495 - 1.476

## Discussion

A variety of treatment regimens have been developed [4-6] and have improved the survival of patients with advanced or recurrent gastric cancer. Currently, combination chemotherapy is considered to be more effective than single-agent therapy. S-1 is an oral antitumor drug that is composed of tegafur, 5-chloro-2,4 dihydroxypyrimidine and potassium oxonate. This drug was designed to enhance the efficacy and reduce the gastrointestinal toxicity of tegafur, a pro-drug of fluorouracil [17-19]. S-1 mono-therapy reportedly achieved a response rate of 45% and 2-year survival rate of 17% [18,20]. In the SPIRITS trial [8], the combination of S-1 and CDDP showed encouraging results as compared to S-1 alone, with response rates of 54% to 31% and OS of 13 months to 11 months. However, the results of the GC0301/TOP 002 (S-1 vs S-1 + CPT-11) revealed that OS with combination therapy did not significantly exceed that with mono-therapy [21]. Other agents for use in combination with S-1, such as taxans, should also be evaluated.

The main rationales for combination treatment with docetaxel and S-1 were synergistic antitumor activity *in vivo *and lack of overlapping toxicities [22]. We previously demonstrated the mechanisms underlying the synergistic effects of docetaxel with S-1 [23]. The expressions of thymidylate synthase and dihidropyrimidine dehydrogenase were decreased and that of orotate phosphorybosyl transferase was increased when docetaxel was administered in combination with S-1. In addition, in recent retrospective and phase I/II study [13,14,24], the combination therapy demonstrated promising results for highly activity and manageable toxicity as first-line regimen for advanced or recurrent gastric cancer.

In this study, combination therapy with docetaxel and S-1 showed good clinical activity with acceptable toxicity in patients with advanced or recurrent gastric cancer. The overall response rate was 52.4%, median TTP 6.5 months, and median OS 15.1 months. The major toxicities were leucopenia (52.3%), alopecia (46.5%), neutropenia (45.3%) and anorexia (41.8%), respectively. Grade 3 or 4 hematologic toxicities included neutropenia (36.0%), leucopenia (31.7%), febrile neutropenia (4.7%) and anemia (1.2%), which occurred in 55.6% (40/72) within three cycles. However, the hematological and non-hematological toxicities were both tolerable, except in one case which died due to Grade 4 neutropenia followed by sepsis, and most subjects could be treated as outpatients. This present results were compatible with those of a previously reported Phase I/II study. Herein, we also found the tumor response to be a prognostic factor indicating increased OS, while other independent factors, such as performance status, disease status and histology metastatic sites, did not affect survival. Second-line chemotherapy also didn't contribute to the favorable OS in this study. There is no established second-line chemotherapy for gastric cancer, but some randomized phase II or III study are now ongoing, such as JACCRO GC-05: the romdomized phase II/III study comparing CPT-11 monotherapy with the S-1/CPT-11 combination for S-1 refractaory gastric cancer. Based on these promising results, a phase III study (JACCRO GC03 study) [25] comparing S-1 alone versus the combination of docetaxel and S-1 has been launched. This is a prospective, multicenter, multinational, randomized study of patients with advanced gastric cancer. The primary objective of the study is to compare median OS with the combination therapy (docetaxel and S-1) to that in the control arm (S-1 alone). In total, 638 patients were enrolled (the original goal was 628 patients, 314 in each treatment arm), and the final results will be reported in 2010. Depending on the results of the GC03 study, this combination regimen may become a first-line standard therapy for patients with advanced or recurrent gastric cancer.

In conclusion, our retrospective study demonstrated that the docetaxel and S-1 combination has good clinical activity with acceptable toxicity when administered as a first-line treatment for patients with advanced or recurrent gastric cancer.

## Competing interests

The authors declare that they have no competing interests.

## Authors' contributions

KT carried out the studies. TS, NT, and HY participated in its design and coordination and helped to draft the manuscript. KY conceived of the study and participated in its design and coordination. HO, chief of our institution helped to draft the manuscript and revised it critically. All authors read and approved the financial manuscript.
